# The Operative Treatment of Internal Piles

**Published:** 1907-10-12

**Authors:** 


### THE OPERATIVE TREATMENT OF INTERNAL PILES.

What is an internal pile? It is an elongated, purple swelling lying with its long axis along the rectal wall. It is caused by obstruction to the venous return from the hæmorrhoidal plexus of veins, due in the great majority of cases to habitual constipation. Some people appear, however, to be born with a hereditary tendency to hæmorrhoids, and suffer from them even in early youth without any constipation to account for their presence.

This article is not concerned with the palliative treatment of hæmorrhoids; it will be assumed that this has been attempted and has failed, and that no contra-indication to operation such as morbus cordis, pregnancy, cirrhosis of the liver, or carcinoma of the rectum is present. The last of these is frequently overlooked. All patients of middle age complaining of piles coming on then for the first time should be carefully examined for carcinoma of the rectum, with the sigmoidoscope if necessary. Many errors of diagnosis would be avoided if this were done systematically.

### The Preparation of the Patient.

In all rectal cases the patient must be most carefully prepared beforehand, so that the bowel is completely empty at the time of operation. Mr. Allingham used to advise giving five grains of pil. hydrarg every night for three or four nights. On the morning of the day before the operation an ounce of castor oil should be administered, and on the morning itself the rectum should be emptied with a soap-and-water enema.

### The Position of the Patient.

The patient should lie on his right side at the edge of the table, with his knees well drawn up. The assistant should stand with his back to the patient's head; his right hand holds up the patient's left buttock and his left is free to assist the surgeon, who should be seated. It is not at all advisable to operate on a patient in the lithotomy position, because the pressure on the abdomen which this causes makes the hæmorrhoidal veins turgid, and what is only a temporary congestion may be mistaken for a hæmorrhoid.

### The Operation.

Of all the operations which have been or are now in vogue for hæmorrhoids removal by ligature is the simplest and the most efficient. The sphincter is gradually and firmly dilated. This may seem a small point, but it is one which makes a great difference to the patient: for, if the sphincter is well dilated, there is rarely much pain after the operation. Moreover, the manœuvre enables the surgeon to define more easily the piles which he intends to remove. However many hæmorrhoids there may appear to be on examination, it is wise never to remove more than three, or at most four, of them. If more are taken away there is a great danger of the patient's suffering from stricture of the rectum, as the result of the contraction of cicatricial tissue, and this is a most troublesome complication. When it is determined which piles are to be removed, they should be drawn down and forceps applied.

In the writer's opinion the ordinary pile forceps sold for this purpose by instrument makers are not as efficient as Spencer Wells' forceps, rather larger than the ordinary size. It is well to put a pair of forceps at once on each of the piles to be removed. If only one is drawn down and operated on at a time, it may be difficult subsequently to recognise, on account of the hæmorrhage, the others which have been destined for removal. The assistant then lifts the forceps and a cut is made with scissors in the sulcus between the skin and mucous membrane extending a little way up the bowel, so the pile is left hanging by an isthmus of skin and mucous membrane. The isthmus is then surrounded with very stout silk, which is tied as tightly as possible. A small portion should be snipped off the apex of the pile; this avoids the possibility of its becoming tense and œdematous afterwards.

The Spencer Wells' forceps is now removed, and the ligature is cut off so that about three or four inches of it is left *in situ*. Each of the piles is treated in a similar fashion. The piles and ligatures are now returned into the rectum, and a strip of gauze with vaseline is inserted to keep them in position, and to lessen the patient's discomfort. A morphia suppository may also be inserted, if desired, although its power as a local analgesic is somewhat doubtful.

### Risks of the Operation.

If properly performed the operation should be devoid of all danger. Occasionally, however, the ligatures slip, and serious hæmorrhage may occur; in this case it is wiser to give another anæsthetic and find the bleeding-point, and either apply another ligature or leave a Spencer Wells' clip *in situ* than to attempt to arrest the hæmorrhage by plugging the rectum with sponges on a string or a petticoated tube. Another possible danger is the occurrence of septic pylephlebitis. This should never occur if the operation has been aseptically performed.

### After Treatment.

The bowels should not be opened before the fourth day. The best aperient to use is castor oil, one ounce of which should be given on the evening of the third or fourth day. Up to this time the diet should be quite light. After this date it may be increased gradually, and the bowels should be kept open by means of a gentle saline purge daily. The ligatures should come away between the seventh and tenth days of convalescence. If after a fortnight one or more of them still remains attached, it means that they were not tied sufficiently tightly at the time of operation. They generally, however, come away eventually. The patient should be kept in bed for at least seventeen days.

